# Molecular analysis of a consanguineous Iranian polycystic kidney disease family identifies a *PKD2* mutation that aids diagnostics

**DOI:** 10.1186/1471-2369-14-190

**Published:** 2013-09-08

**Authors:** Reza Vazifehmand, Sandro Rossetti, Sassan Saber, Hamid Reza Khorram Khorshid, Peter C Harris

**Affiliations:** 1 Department of Molecular Pathology, Massoud Laboratory, Tehran, Iran; 2 Young Researchers Club, Islamic Azad University, Rasht, Iran; 3 Division of Nephrology and Hypertension, Mayo Clinic, 200 First Street SW, Rochester, MN 55905, USA; 4 Department of Internal Medicine, Shariati Hospital, Tehran University of Medical Sciences, Tehran, Iran; 5 Genetic Research Centre, University of Social Welfare and Rehabilitation Sciences, Tehran, Iran

**Keywords:** ADPKD, PKD2, Molecular diagnostics

## Abstract

**Background:**

Polycystic kidney diseases (PKD) are a group of monogenic disorders that are inherited dominantly (autosomal dominant PKD; ADPKD) or recessively, including, autosomal recessive PKD (ARPKD). A number of recessive, syndromic disorders also involve PKD but have a range of pleiotropic phenotypes beyond the kidney, and are enriched in consanguineous families.

**Case presentation:**

We describe here a consanguineous Iranian pedigree in which PKD was diagnosed in four generations, but also included cases with additional abnormalities, including mental retardation. We employed molecular screening to reveal the etiology of the PKD. Since the PKD seemed to be dominantly inherited, molecular diagnostics was performed by direct sequencing of the ADPKD genes, *PKD1* and *PKD2*. Clinical and imaging data was collected on family members. The sequence analysis revealed a *PKD2* single base-pair deletion, c.1142delG, and segregation was demonstrated in 16 PKD patients from different branches of the family. In keeping with other reports, the PKD2 phenotype in this family was overall mild, and characterized by conserved kidney function, although 12 cases had some evidence of renal insufficiency. Several younger mutation carriers had borderline or no clinical characteristics of ADPKD, while a patient that required a renal transplant at 14 y did not have the *PKD2* mutation.

**Conclusions:**

The molecular analysis of an Iranian family showed that the PKD was due to a *PKD2* mutation. The identification of the causative mutation allowed an accurate diagnosis in a number of individuals with equivocal imaging data. Consequently, these patients could be followed appropriately as at-risk individuals. In addition, the PKD2 diagnosis ruled out a syndromic form of PKD as the cause of the additional phenotypes in the family.

## Background

Polycystic kidney diseases (PKD) are a group of monogenic disorders characterized by cyst development in the kidney, but also often have extrarenal manifestations [1]. This group of disorders has been linked to defects in the functioning of primary cilia and therefore are termed ciliopathies [2]. The most common form of the disease (incidence 1/500-1000) is autosomal dominant PKD (ADPKD) that is typically a late onset disease characterized by progressive cyst development and often resulting in end-stage renal disease (ESRD) [3]. Clinically significant extrarenal manifestations include severe polycystic liver disease and an increased prevalence of intracranial aneurysms. ADPKD is genetically heterogeneous with two genes known, *PKD1* and *PKD2*[4,5]. PKD1 accounts for ~85% of clinical cases and is associated with more severe disease, with ESRD at 54.3 y compared to 74 y for PKD2 [6]. Recent data from Cornec-Le Gall also indicated a significance difference in age at ESRD between PKD1 cases with truncating compared to non-truncating mutations (55.6 y vs. 67.9 y, respectively) [7]. Diagnostics is usually possible by renal imaging with specific criteria determined for a positive diagnosis by ultrasound [8,9], but molecular diagnostic screens can also be helpful to determine the gene involved and to identify at-risk individuals [10,11]. In particular, knowing the gene and mutation type can be of prognostic value (see above), and in rare cases where there is interest, facilitate preimplantation genetic diagnostics.

The most common recessive form of PKD, autosomal recessive PKD (ARPKD; incidence 1:20,000), is most often a neonatal onset disorder and associated with significant neonatal demise; although adult presentation of the disease associated with at least one hypomorphic allele is increasingly recognized [1,12,13]. A number of other recessively inherited, syndromic forms of PKD have additional disease manifestations likely associated with ciliary dysfunction [2]. These diseases include the lethal Meckel syndrome, Joubert syndrome, Bardet Biedl syndrome and orofacial digital syndrome. Extrarenal manifestations range from liver, eye and digital defects to central nervous system abnormalities, including mental retardation. These rare disorders are enriched in consanguineous populations.

We describe here a complex Iranian family with multiple consanguineous relationships that manifests PKD, but also a number of other abnormalities, including heart defects, renal agenesis and mental retardation. Molecular testing of the ADPKD genes identified the molecular defect; highlighted variability associated with this mutation, and diagnosed at-risk individuals with negative or equivocal renal imaging data.

## Patients and methods

The study was approved by the Ethics Committee at the Islamic Azad University (8888117001) and the Mayo Clinic IRB (285–00). Written informed consent was obtained from all patients for publication of this Case report. A copy of the written consent is available for review by the Editor of this journal. Clinical information was obtained by review of the clinical records and by interviewing the patients. Clinical data on kidney function (serum creatinine) and ultrasound imaging data to determine kidney size, number of cysts and the presence of renal stones was obtained for at-risk individuals, as available. The pedigree was drawn employing the Cyrillic program. Blood samples were collected from 25 family members for DNA isolation by standard salting out methods. The genomic DNA of the proband (V:16) was PCR amplified for all the coding exons of the *PKD1* and *PKD2* genes following previously published protocols [14-16]. Mutation analysis was performed by bidirectional sequencing on PCR-amplified products of all the coding exons for both the *PKD1* and the *PKD2* gene. Chromatograms were analyzed using the software Mutation Surveyor (SoftGenetics Inc.). Segregation analysis for the detected disease-associated mutation was performed in all available family members.

## Case presentation

**Figure 1:**
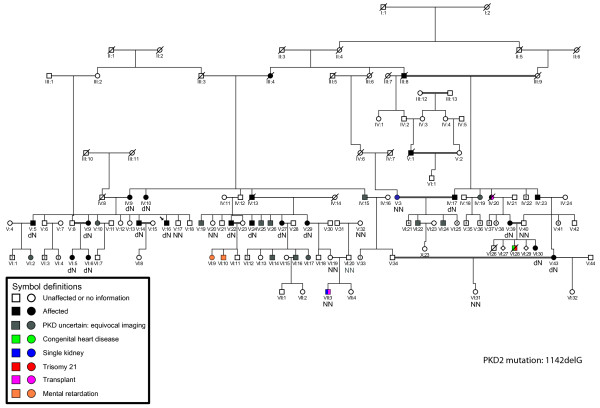
**Pedigree of the large Iranian family showing the PKD (Affected) and other phenotypes found in the family (see key for details).** The genotype for the *PKD2*: 1142delG mutation is indicated as N=normal or, d=deleted.

**Figure 2:**
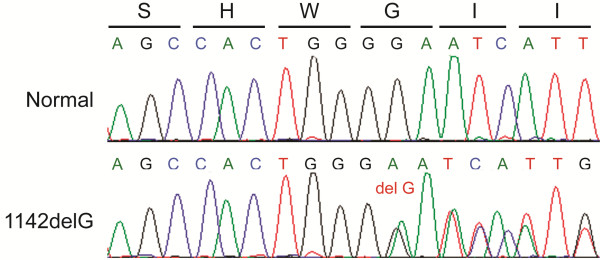
**Sequence chromatograph showing the deletion of G at position 1142 in exon 5 of the *PKD2 *gene.** Note that the G is in a run of four Gs that may have contributed to this frame-shifting mutation at this site.

This family first came to our notice when the proband (V:16) underwent abdominal ultrasound analysis at 30 y and a single, large cyst was detected in each kidney. Although these results did not meet the Ravine criteria for an ADPKD diagnosis, taking a family history revealed evidence of other family members with renal cystic disease (Table 1). Careful tracing of the family, plus renal ultrasound analysis and serum creatinine measurements, revealed a large family in which 125 individuals could be traced with at least 30 having some evidence of renal cystic disease, in four generations (Figure 1). The family had at least nine consanguineous couples, usually first cousins, and also cases with mental retardation, congenital heart disease renal agenesis and early onset ESRD. Although the consanguinity and range of extra-renal manifestations (some similar to those found in ciliopathies) suggested that recessive inheritance may be important, the PKD seemed to be inherited mainly in a dominant fashion. We, therefore, screened the ADPKD genes, *PKD1* and *PKD2,* by sequence analysis for mutations in the proband. This screening revealed a *PKD2* deletion, c.1142delG: p.G381fs71X as the only likely disease causing mutation (Figure 2). Segregation analysis in the subjects where DNA was available showed that 16 had the *PKD2* mutation indicating that this is a PKD2 family. PKD2 is typically a much milder disease than PKD1 and this is reflected in this family where many individuals had rather few cysts and normal renal function (Table 1). Exceptions were IV:20 who had a renal transplant at 50 y and V:1 who started dialysis at 54 y. Other individuals with marked renal insufficiency were V:29 with an eGFR of 20 ml/min/1.73 m^2^ at 54 y, V:39 with an eGFR of 31 ml/min/1.73 m^2^ at 38 years and two older family members with eGFR of 27 (IV:10) and 39 ml/min/1.73 m^2^ (IV:17) at 75 y and 63 y, respectively. Several younger individuals shown to inherit the *PKD2* mutation had cyst numbers below the threshold for diagnosis by ultrasound and in at least one case at 20y (VI:30) no renal or hepatic cysts were detected. Kidney stones were commonly found in affected cases and are a known complication of ADPKD.

**Table 1:** Clinical details of affected cases and others with unusual phenotypes

**Patient No.**	**Sex**	**Age (y)+**	**Renal Function (Serum Creatinine; mg/dl)**	**Kidney Length^ (mm)**		Notable Kidney Features from Imaging*		**Other Findings**	**Genetic results**
				**Right**	**Left**	**Right**	**Left**		
**Affected**									
III:4	F	56	N/A	N/A	N/A	N/A	N/A	Dead; PKD	N/D
III:8	M	63	N/A	N/A	N/A	N/A	N/A	Dead; PKD	N/D
IV:9	F	67	1.4	113	125	LC 10x6 mm	Normal		1142delG
IV:10	F	75	1.8	108	135	Multiple cysts	9 mm stone		1142delG
IV:13	M	77	Dialysis (75 y)	105	100	LC 22x10 mm	11 mm stone in middle caulis	Dead; PKD	N/D
IV:17	M	63	1.8	118	110	LC 15x8 mm	9 mm stone		1142delG
IV:20	F	53	Tx (50 y)	150	160	Multiple cysts	Multiple cysts	Dead; PKD	N/D
IV:23	M	50	0.9	115	105	4 mm stone	LC 15x8 mm		N/D
V:1	M	54	Dialysis (54 y)	N/A	N/A	N/A	N/A	Dead; PKD	N/D
V:5	M	42	1.1	125	110	LC 15x8 mm	3 mm stone		1142delG
						2 mm stone in middle caulis			
V:9	F	38	1.3	100	95	LC 23x18 mm	9 mm stone in middle caulis		1142delG
						3.5 mm stone in middle caulis			
V:14	M	32	0.9	105	105	Multiple cysts	Stone 3 mm		1142delG
V:16 (proband)	M	30	1.2	105	100	1 cyst, 12x12 mm	1 cyst, 27x22 mm		1142delG
V:22	M	46	1.2	118	110	3 mm stone	1 cyst, 13x13 mm		1142delG
V:24	M	35	0.9	105	107	Normal	2 mm stone		1142delG
V:27	F	49	1.3	108	115	Normal	3.5 mm stone		1142delG
							LC 3x3 mm		
V:29	F	54	2.5	105	110	1 cyst 10x10 mm	1 cyst, 8x8 mm	1 liver cyst	1142delG
							3 mm stone		
V:39	F	38	1.8	160	170	Multiple cysts	Multiple cysts		1142delG
							4.5 mm stone in interlobular region		
V:43	F	37	0.7	112	124	Normal	1 cyst, 13x13 mm	22x23 mm liver cyst	1142delG
VI:5	F	20	0.7	95	90	1.5 mm stone	Normal		1142delG
VI:6	F	16	0.7	108	109	Normal	3 mm stone		1142delG
VI:30	F	16	0.8	108	98	Normal	Normal		1142delG
**PKD suspected but not proven**									
IV:15	M	62	0.8	105	100	LC 20x10 mm	9 mm stone in middle caulis		N/D
						3.5 mm stone in middle caulis			
IV:19	F	51	1.5	108	105	1 cyst, 12x8 mm;10 mm stone	11 mm stone in middle caulis		N/D
V:10	F	41	1.2	108	105	1 cyst, 10x5 mm	Normal		N/D
V:19	M	40	1.1	112	108	1 cyst, 6x6 mm	Normal		N/D
V:25	M	32	0.8	100	105	1 cyst, 6x6 mm	Normal		N/D
V:26	M	26	0.8	105	100	2 mm stone	1 mm stone		N/D
VI:2	F	14	0.8	95	107	Normal	1 cyst, 5x4 mm; 1.5 mm stone		N/D
VI:14	M	24	1.3	107	105	2 mm stone	1 cyst, 7x6 mm		N/D
VI:16	M	24	1.7	110	112	Normal	1 cyst, 8X10 mm		N/D
VI:17	F	17	0.8	95	95	1 stone	Normal		N/D
VI:22	M	45	1.4	107	105	1 cyst, 8x5mm	2.6 mm stone		N/D
VI:24	M	33	1.1	112	100	1 cyst, 6 mm	Normal		N/D
**Unaffected**									
V:3	F	53	0.9	105	---	Normal	Congenitally absent		1142G
V:17	M	39	0.9	110	100	Normal	Normal		N/D
V:32	F	64	3.59	100	95	43 mm stone, hydronephrosis	6.7 mm stone		1142G
VI:9	F	20	0.7	100	96	Normal	Normal	Non Syndromic Mental Retardation,	N/D
								Normal Karyotype	
VI:10	M	16	0.7	108	109	Normal	Normal	Non Syndromic Mental Retardation	N/D
								Normal Karyotype	
VI:19	F	34	0.8	95	105	Normal	Normal		1142G
VI:20	M	46	0.7	100	105	Normal	Normal		1142G
VI:28	M	?	N/A	N/A	N/A	N/A	N/A	Congenital heart disease, Down syndrome; Dead	N/D
VII:3	M	17	Tx (14y)	85	---	No cysts	Congenitally absent	Reflux Nephropathy	1142G

*LC, largest cyst; ^*N/E*, not enlarged; *N/D*, not determined; *N/A*, not available; + = age last imaging data available.

A risk in such a consanguineous family is that both parents will be affected in which case one in four of their pregnancies would be homozygous for the *PKD2* mutation. *Pkd2*^*−/−*^ mice are embryonic lethal, dying by 14.5d of gestation, and it is assumed in humans, but never proven, that having two fully penetrant *PKD2* mutations is incompatible with life. To further determine the significance of the *PKD2* mutation in this family, we analyzed a number of family members with unusual manifestations, including kidney agenesis, early onset ESRD or other extra-renal manifestations sometimes related with syndromic PKD, to see if the *PKD2* mutant allele could be playing a role. IV:17 is affected and second cousin to his partner (V:3), who had just a single kidney, but genetic analysis showed she did not have the *PKD2* mutation. V:40 is married to his affected first cousin (V:39) and they had two children who died at a young age (one with Down syndrome and congenital heart disease), but again the father did not have the *PKD2* mutation. VII:3 had a solitary kidney and ESRD requiring a renal transplant at 14y, although no cysts were detected. His maternal grandmother (V:29) had renal insufficiency due to PKD (Table 1) and large kidney stones and the paternal grandmother (V:32) had kidney stones and hydronephrosis. However, genetic analysis showed that neither of his parents (VI:19 nor VI:20) or the paternal grandmother had the *PKD2* mutation, and so the renal failure was not PKD related and most likely due to reflux nephropathy. The two sibs with mental retardation (VI:9 and VI:10) are at risk for PKD2 as the father is affected, but neither had evidence of cysts at 20y and 16y, respectively. Unfortunately, no samples were available to test if they had the *PKD2* mutation.

## Conclusions

We describe here an Iranian family with multiple consanguinity loops with over 30 individuals with PKD. Despite the complex structure of the family, by molecular testing we have been able to show that the PKD is due to a truncating mutation to *PKD2*. This firm diagnosis rules out that syndromic forms of PKD that are enriched in consanguineous populations are playing a role and showed that renal failure in one child was not related to the *PKD2* mutation.

Considerable phenotypic variability was seen in the family both in terms of renal survival, kidney dimensions and the occurrence of kidney stones. However, it is well document that there can be considerable intra-familial variability in ADPKD, indicating that other genetic and environmental factors significantly influence the phenotype [17].

Renal ultrasound and other imaging methods are a reasonably reliable method to diagnose ADPKD in at-risk adults. However, ultrasound is less reliable in the milder PKD2, especially in younger individuals, as illustrated in this family with several cases with equivocal imaging data, showing even with the recent ultrasound criteria that there is a significant false negative rate, especially when state-of-the-art ultrasound equipment is not employed [8,18]. Molecular diagnostics in ADPKD requires some considerable effort because of the involvement of the large and complex *PKD1* gene, as well as *PKD2*, and because of the high level of allelic heterogeneity meaning that both genes need to be fully sequenced to identify the disease gene [10,19]. Commercial testing is therefore expensive. However, this family illustrates the useful role for molecular diagnostics in ADPKD. PKD2 was identified as the disease, and once the disease mutation has been detected the rest of the family can be readily and inexpensively screened, with unequivocal diagnostic data obtained. In this family, this has helped resolve the etiology in some patients with unusual phenotypes and identified those at risk for developing ADPKD even if no clinical signs are yet present. This means precious resources can be concentrated on following these individuals, with prompt treatment of hypertension, urinary tract infections, kidney stones and other complications [11]; and not those unaffected. Although molecular diagnostic is not required in every suspected ADPKD family it does have a valuable part to play in some complex families.

## Abbreviations

PKD: Polycystic kidney disease; ADPKD: Autosomal dominant PKD; ARPKD: Autosomal recessive PKD; ESRD: End-stage renal disease; LR-PCR: Long range PCR.

## Competing interests

The authors declare that they have no competing interests.

## Authors’ contributions

RV conceived the study, collected DNA samples and clinical data and helped write the paper; SR did the molecular studies and helped write the paper; SS collected the biochemical information and edited the paper; HRKK helped collect the clinical data and edited the paper; and PCH oversaw the study and prepared the final draft of the paper. All authors read and approved the final manuscript.

## Pre-publication history

The pre-publication history for this paper can be accessed here:

http://www.biomedcentral.com/1471-2369/14/190/prepub
